# Description of a ‘plankton filtration bias’ in sequencing-based bacterial community analysis and of an Arduino microcontroller-based flowmeter device that can help to resolve it

**DOI:** 10.1371/journal.pone.0303937

**Published:** 2024-05-28

**Authors:** Corentin Fournier, Alexander Fiedler, Maximilian Weidele, Harald Kautz, David Schleheck

**Affiliations:** 1 Department of Biology, Microbial Ecology and Limnic Microbiology, Limnological Institute, University of Konstanz, Konstanz, Germany; 2 Scientific Engineering and Manufacturing Services, University of Konstanz, Konstanz, Germany; University of Kalyani, INDIA

## Abstract

Diversity studies of aquatic picoplankton (bacterioplankton) communities using size-class filtration, DNA extraction, PCR and sequencing of phylogenetic markers, require a robust methodological pipeline, since biases have been demonstrated essentially at all levels, including DNA extraction, primer choice and PCR. Even different filtration volumes of the same plankton sample and, thus, different biomass loading of the filters, can distort the sequencing results. In this study, we designed an Arduino microcontroller-based flowmeter that records the decrease of initial (maximal) flowrate as proxy for increasing biomass loading and clogging of filters during plankton filtration. The device was tested using freshwater plankton of Lake Constance, and total DNA was extracted and an 16S rDNA amplicon was sequenced. We confirmed that different filtration volumes used for the same water sample affect the sequencing results. Differences were visible in alpha and beta diversities and across all taxonomic ranks. Taxa most affected were typical freshwater Actinobacteria and Bacteroidetes, increasing up to 38% and decreasing up to 29% in relative abundance, respectively. In another experiment, a lake water sample was filtered undiluted and three-fold diluted, and each filtration was stopped once the flowrate had reduced to 50% of initial flowrate, hence, at the same degree of filter clogging. The three-fold diluted sample required three-fold filtration volumes, while equivalent amounts of total DNA were extracted and differences across all taxonomic ranks were not statistically significant compared to the undiluted controls. In conclusion, this work confirms a volume/biomass-dependent bacterioplankton filtration bias for sequencing-based community analyses and provides an improved procedure for controlling biomass loading during filtrations and recovery of equivalent amounts of DNA from samples independent of the plankton density. The application of the device can also avoid the distorting of sequencing results as caused by the plankton filtration bias.

## Introduction

The advent of ‘meta-omics’ technologies has enabled environmental microbial ecology research to go a huge step forward in the disentanglement of the complexity of the highly diverse and dynamic microbial communities on this planet. This also applies to the plankton communities in pelagic marine and freshwater ecosystems, particularly the microscopic, so-called ‘nanoplankton’ (herein, the organismal group with 5–180 μm in diameter) as representation of the, predominantly, phytoplankton, and the ‘picoplankton’ (herein, the organismal group with 0.2–5 μm in diameter) as representation of the, predominantly, single-cell bacterioplankton, which each on their own and by their interactions are playing key roles for functioning and stability of these ecosystems [1–4]. Total DNA extraction of nano- and picoplankton communities of environmental samples and PCR amplicon sequencing of phylogenetic markers (e.g., fragments of 16S and 18S rRNA genes or other markers) enables the examination of diversity and relative abundance of its community members and identification of the active members within these communities. Metagenome sequencing allows for an evaluation of the genetic and functional repertoire encoded in the community members [5–7], while metatranscriptomics as well as metaproteomics allow for a detection of the genes and functional traits that are actually expressed under any particular environmental condition [8, 9]. However, the high sensitivity of these modern techniques and the long methodological pipelines involved, are accompanied by increased vulnerability to biases that may be introduced by each methodological step, e.g., for PCR amplicon sequencing through DNA extraction, primer choice, PCR [10], DNA-template dilution [11], as well as library preparation for sequencing [10, 12, 13].

For analyzing nano- and picoplankton communities *via* meta-omics methods, a key step is the sampling process, since cellular biomass needs to be isolated from the water samples in sufficient quantities to allow for, e.g., extraction of total DNA or RNA. Filtration is the principal process most commonly used for collecting nano- and picoplankton biomass from environmental samples. Usually, a pre-filtration is applied to remove zooplankton and other larger particles using a pore-size filter in the range of 100–200 μm [14, 15]. The sample is then passed through either one or a series of different smaller pore size filters to collect the nano- and/or picoplankton size classes. An intermediate pore-size filter, e.g. 5 μm diameter, can be considered to collect the nanoplankton, i.e., predominantly the eukaryotic (protist) phytoplankton, but also particle-associated and filamentous bacteria, archaea or fungi (collectively, nanoplankton) [5, 16–20]. A subsequent smaller pore-size filter, typically in the range 0.1–0.22 μm diameter, can be considered to collect the remaining single-cell prokaryotic and picoeukaryotic plankton (collectively, picoplankton) [5, 16, 20–22]. Besides many options in choosing filter techniques and pore sizes, also a variety of filter materials is existing, e.g., nylon net, glass fiber, polycarbonate or polyethersulfone membranes, and their different efficiencies in collecting plankton biomass have been subject of discussions [23, 24]. Additionally, the filtration volume can be variable, either in between studies (from 0.3 L up to 25 L) [6, 15, 25], within the same study [25], or in between different types of omics-analyses done on the same water sample [26, 27]. Hence, it is easy to rationalize that using different filtration settings will result in different final outcomes of plankton diversity analyses [28], in addition to the above described biases that may be introduced by the molecular pipelines used.

We questioned whether also the filtration volume of a plankton sample may influence the outcome of the results under a given filtration and community-analysis setting, and if so, in which proportion. Indeed, Padilla *et al*., 2015 have already described a volume-dependent ‘plankton filtration bias’, when they analyzed the picoplankton community structure for an identical water sample (seawater) by 16S rDNA amplicon sequencing in dependence on different filtration volumes used, and their results showed significant differences in the relative abundances of bacterial community members in dependence on the water volume filtered [29]. This prompted us to consider whether the cause of such a volume-dependent filtration bias might indeed be a build-up of a so-called ‘filtration cake’ during the filtration process: that is, the increasing layer of cellular biomass collected during the filtration process might increasingly act as an additional filter, thereby retaining microorganisms that would normally belong into the filtrate and thus onto the subsequent smaller pore-size filter. We further considered whether one way of avoiding, or of normalizing, such a sample volume /plankton density-dependent filtration bias may be the collection of each the same amount of total biomass on each filter, with adjusted filtration volumes in dependence on the plankton density of the samples.

**Fig 1:**
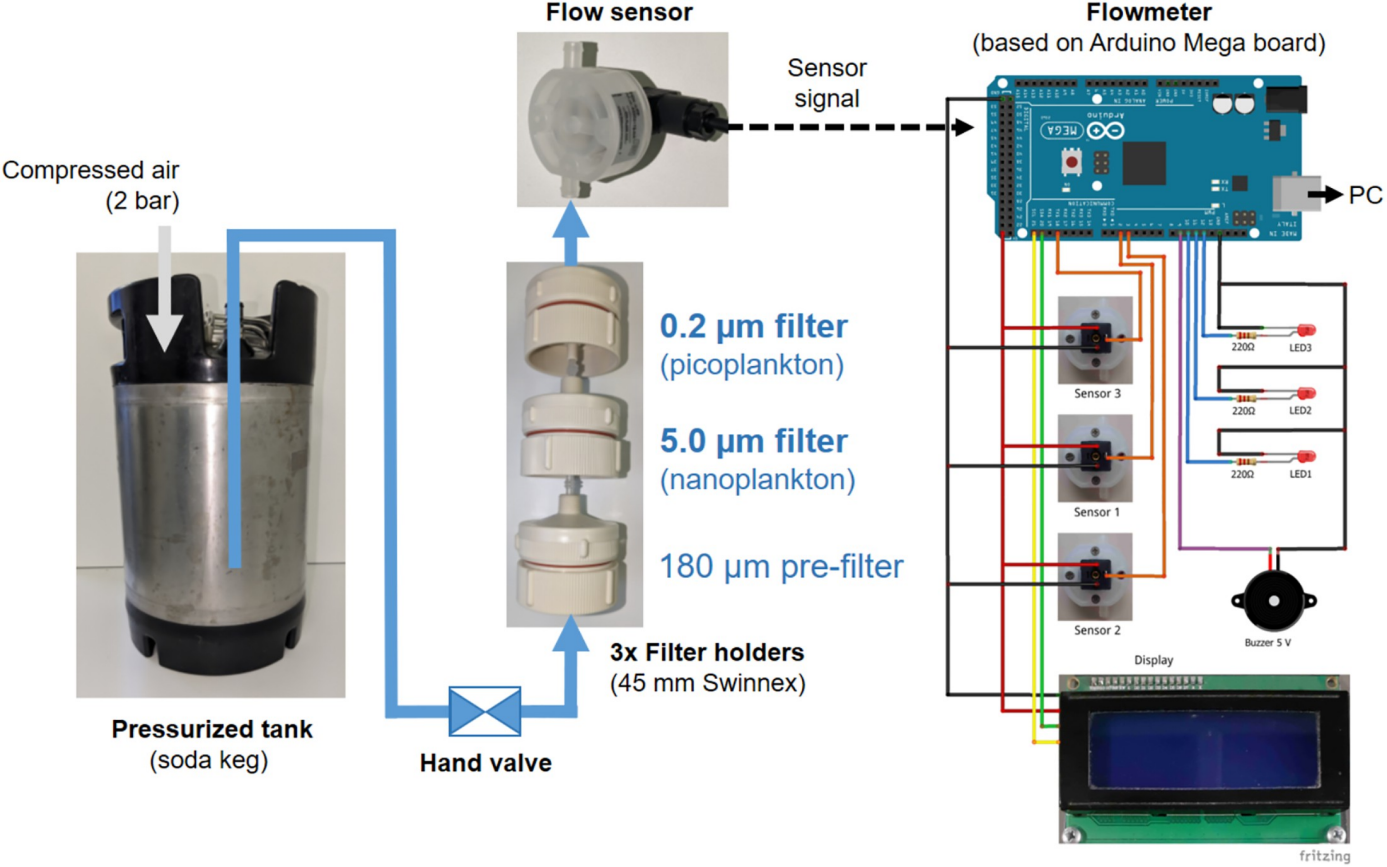
Schematic of the filtration setup and flowmeter device used in this study. We used overpressure filtration for plankton size-class filtration with three filters in series, (*i*) a 180-μm nylon net pre-filter to remove zooplankton and larger particles, and (*ii*) a 5.0-μm and (*iii*) a 0.2-μm pore-size polycarbonate membrane filter, in order to collect the nanoplankton and picoplankton, respectively, for DNA extraction. The flowrate of the filtrate was monitored continuously during the filtration, by using a flow sensor and a flowmeter device based on an Arduino board. The Arduino was programmed to record the initial, maximal flowrate (F_m_) when a filtration had been started with new filters, and to monitor the continuous decrease of flowrate caused by increasing biomass loading and clogging of the filters. Once a pre-defined (programmed) minimal flowrate threshold (F_t_) has been reached, the Arduino was configured to give an optic (LED) and acoustic (beeper) alarm to the operator, so that the filtration could be stopped manually using a valve, for example, each at F_t_ = 50% F_m_ across water samples with variable plankton densities. The flowrate data was also streamed to a PC for recording (see Fig 2A). Note that the illustration is not to scale. Details on components and operation of the filtration monitoring setup can be found in the Material and methods section, and details on the Arduino configuration and the Arduino code at Github (https://github.com/Uni-Konstanz-WWE/UKon_Plankton_Filtration_Flowmeter). The Arduino electronic wiring was illustrated using the software tool Fritzing (https://fritzing.org/).

In order to explore these considerations experimentally, and as reported in this communication, we constructed an Arduino-microprocessor based flowmeter device (Fig 1) that allows to record the decrease of initial, maximal flow rate during the filtration process over time as a proxy of increasing filter loading (clogging). It also allows to stop the filtrations each after the same decrease of maximal flowrate (i.e., at the same minimal-flowrate threshold) and, hence, at about the same biomass (particle) loading.

We tested the device using plankton samples of Lake Constance by sequentially collecting the nano- and picoplankton size classes (after 180-μm pre-filtration) onto and 5.0-μm and 0.2-μm pore-size membrane filters, respectively (see Fig 1). Then, the single-cell bacterial community structures represented on the 0.2-μm pore size filter were examined by total DNA extraction and 16S rRNA gene amplicon sequencing. Our first objective was to confirm the volume-dependent filtration bias as revealed by Padilla *et al*., 2015 (see above). Second, we tested another Lake Constance water sample at two different plankton densities, i.e., the original, undiluted sample and a three-fold diluted sub-sample, when using our flowmeter to stop the filtration each at the same flowrate-threshold. Hence, the second experiment was conducted to evaluate whether the flowmeter device may be helpful in normalizing a density/volume-dependent filtration bias.

## Materials and methods

### Study sites and sampling campaign

Lake Constance is a deep (maximal depth, 251 m) oligotrophic pre-Alpine lake situated in the northern part of the Alps (47°35´N 9°28´E). The lake is composed of three water bodies called *Obersee* (Upper lake), *Untersee* (Lower lake) and *Seerhein* (Lake Rhine); the Lake Rhine is connecting the Upper Lake to the Lower Lake. In this study, samples were taken at the routine sampling site *Wallhausen* situated in the *Überlinger See* (47.7571°N 9.1273°E), a fjord-like northwestern arm of the main basin of Upper Lake. No permits were required for accessing the sampling site and for the water sampling. Integrated samples of the epilimnion (0–20 m) were taken by ship using an integrating water sampler (model IWS II, Hydro-Bios, Germany). The samples used for the first experiment were collected on 08. November 2017 and the samples for the second experiment on 14. May 2019. The water samples were transferred in stainless steel barrels (soda kegs) and immediately filtered (see below).

### Filtration using a self-constructed Arduino-based monitoring device (flowmeter)

Filtrations were carried out directly on the ship. A pressure barrel (soda keg with appropriate plug-in couplings) containing the water sample was connected to compressed air and an overpressure of 2 bar was applied. The barrel was connected *via* a hand valve (Riegler, Germany) and PVC tubing (4 mm inner diameter) to a series of three in-line filter holders (Swinnex 47 mm filter holder; Millipore), carrying (*i*) a 180-μm filter (hydrophilic nylon net, 47 mm diameter; Millipore) to remove zooplankton and larger particles, and (*ii*) a 5.0-μm and (*iii*) a 0.2-μm polycarbonate membrane filter (Isopore, 47 mm diameter; Millipore) to collect the nanoplankton and picoplankton, respectively. The sets of three Swinnex filters were connected with Luer connectors (male/male, Carl Roth, Germany) and the filter sets to the PVC tubings via Luer-hose connectors (male/4 mm hose inner diameter, Carl Roth, Germany). At the outlet of the filter set, a mini-flowmeter (model FCH-m-PP –3.0 LPM 82202739; B.I.O-TECH, Vilshofen, Germany) was connected, from which pulses were counted and flowrates calculated (in ml/min) by a programmed Arduino I/O device. The repeatability of the frequency response of the sensors was +/- 0.5% at the same operating conditions. The calibration factor had to be set directly in the Arduino program. The variability of total filtration volume determination by the device compared to the flow-through volume as determined with a measuring cylinder as reference was smaller than +/- 3% when tested without filters in the laboratory. During filtrations in the field (on the ship) using the sets of three filters (Fig 1), it was usually not more than +/-10% variation; if it was higher, e.g. due to filter leakage, the filtration was repeated with a fresh set of filters.

A detailed description of the most basic, low-cost, open-source version of this Arduino flowmeter device, and its electronic components, a circuit diagram, and the software used, is available at GitHub, https://github.com/Uni-Konstanz-WWE/UKon_Plankton_Filtration_Flowmeter. This flowmeter device comprised also an LCD screen that displayed, (*i*) the maximal flowrate (F_m_) recorded right at the start of a new filtration (i.e., directly after the valve had been fully opened), (*ii*) the current flowrate during the filtration (F_c_), and (*iii*) the flowrate threshold (F_t_) at which the filtration has to be stopped; F_t_ is calculated by the Arduino from F_m_ using a threshold factor (e.g., 50% of F_m_), and this threshold factor had to be set directly in the Arduino program. When the flowrate had decreased and F_t_ was reached, the Arduino gave both an optical signal (LED) and acoustic signal (piezo beeper) in order to alert the operator to stop the filtration by manually closing the valve. Optionally, the Arduino was streaming the monitored flowrate to a computer which stored the data in an Excel sheet with graphical display, in order to follow the flowrate on screen at any time during the filtration procedure (Fig 1). Such a basic, low-cost flowmeter device, which can be operated in the field also by battery and without USB connection to a computer, was used for the experiments described in this paper. A much further developed version of the device is currently used for routine sampling of the Lake Constance nano- and picoplankton, comprising six flowmeter channels, a water resistant casing with rechargeable battery, a number pad for adjusting flowrate thresholds and other parameters, and additional I/O ports for data storage on USB stick and for controlling electrically-actuated valves for automation of the filtration procedure.

For the first experiment, we aimed at confirming the observations made by Padilla *et al*., 2015 (see Introduction), but without prior definition of the volumes to be filtered. Instead, we programmed the alarm of the flowmeter for stopping the filtrations when either 66%, 50%, 25% or 10% of the initial, maximal flowrate (F_m_) had been reached, using each the same water sample from Lake Constance (see above); for each threshold, filtrations were done in triplicates (n = 3). For the second experiment, we simulated a change in plankton density, to evaluate whether the flowmeter can indeed be helpful in a normalization of the volume/density-based filtration bias. First, a water sample from Lake Constance was filtered using the flowmeter at a flowrate-threshold of 50% F_m_; the filtration volumes were recorded (590 ml +/- 64 ml). Then, the lake water was diluted 1:3 with autoclaved distilled water and a new series of filtrations (n = 4) was started, using the flowmeter flowrate-threshold of 50%. Another series of filtrations (n = 4) was done ‘volume-controlled’, i.e., until the same volume was reached as for the filtration of the undiluted samples (590 ml). Hence, this gave three sets of samples: undiluted (UD), diluted flowrate threshold filtration (TF1/3) and diluted fixed volume-filtration (VF1/3).

When the filtrations had been stopped, the filter holders were opened and each filter membrane was carefully curled up using two sterile forceps and transferred into a 15 ml conical tube (Eppendorf, Hamburg, Germany) so that the biomass-containing side of the filter was pointing to the inside of the tube. Then, the biomass was immersed each with 3 ml of lysis buffer (50 mM Tris-HCl buffer pH 8.0, 50 mM EDTA, 50 mM EGTA). The tubes were stored on dry ice for the rest of the ship cruise and later in the lab at -20°C in a freezer.

### DNA extraction, PCR amplification and Illumina-amplicon sequencing

The DNA extraction protocol used was adapted from [30] and the JGI bacterial DNA extraction protocol (version 3 by William S., Helene Feil and A. Copeland). After thawing of the samples at room temperature, 200 μl of 0.1- and 1-mm diameter zirconium beads each (Carl Roth, Karlsruhe, Germany) were added to each tube. Then, the tubes were treated in an ultrasonic water bath (Sonorex super RK 510, Bandelin, Germany) for 1 min, followed by 15 min of vortexing at full speed in a horizontal tube holder. In the next step, 150 μl of freshly prepared lysozyme solution (final concentration 2.5 mg/ml) was added, and the tubes incubated for 1 h at 37°C by horizontal shaking at 1,400 rpm (Thermomixer comfort, Eppendorf, Hamburg, Germany); then 315 μl of 10% sodium dodecyl sulfate (SDS) (final concentration, 1%) and 31.5 μl of freshly prepared proteinase K solution (final concentration 500 μg/ml) were added, followed by an incubation of the tubes for 1 h at 55°C in a water bath. After one hour of incubation, proteinase K was added again at the same concentration and the solution incubated again for 1 h at 55°C. Finally, 236 μl of 5 M NaCl solution was added, the solution vortexed, followed by addition of 236 μl of CTAB/NaCl-solution (10% CTAB, 0.7 M NaCl; preheated at 65°C); the solution was again vortexed, and then incubated for 10 min at 65°C. The DNA was purified by phenol-chloroform extraction. Therefore, 1 vol. of phenol/chloroform/isoamyalcohol (25:24:1 v/v %) (Carl Roth, Karlsruhe, Germany) was added, the suspension mixed by vortexing, and the tubes centrifuged for phase separation at 13,000 rcf for 20 min at 4°C. After transfer of the supernatant into a new 15 ml tube, 1 vol. of chloroform/isoamylalcohol (24:1 v/v %) was added, the suspension mixed by vortexing, and the tube centrifuged for phase separation at 13,000 rcf for 20 min at 4°C. The supernatant was transferred into a new 15 ml tube. 0.5 μl of glycogen (Thermo Fisher Scientific, USA) and 0.7 vol. of isopropanol were added to the tube. The solution was mixed well and incubated for 15 min at -20°C. The precipitated DNA was collected by centrifugation at 15,000 rcf for 25 min and 4°C. Isopropanol was removed and the DNA pellet was washed using 500 μl of ice-cold 70% ethanol. After a final centrifugation of 5 min, the supernatant was removed and the DNA dried at air for 5 min. Then, 50 μl of PCR-grade water was added and the DNA dissolved. Finally, the DNA concentration was measured using a Nanodrop 2000c spectrophotometer (Thermo Fisher Scientific, USA) and the quality of the DNA evaluated by agarose gel electrophoresis.

Amplification of the V3-V5 hypervariable regions [31, 32] of the 16S rRNA gene was performed with 0.02 U/ μl of Phusion High Fidelity DNA polymerase, 1X Phusion HF Buffer and 200 μM of dNTPs (New England Biolabs, USA). DNA template was added at a final concentration of 0.12 ng/μl. The primers pair used was 357F and 926R with universal adapter, required for the second PCR, added to their 5’ end. The primer sequences, with universal adapter, are 5´–TCGTCGGCAGCGTCAGATGTGTAT AAGAGACAG-CTCCTACGGGAGGCAGCAG–3´ for 357F and 5´–GTCTCGTGGGCTC GGAGATGTGTATAAGAGACAG-CCGYCAATTYMTTTRAGTTT–3´ for 926R [33–35], at a final concentration of 0.5 μM each. The following PCR program was used on a T100 Thermal cycler (Bio-Rad, USA): first denaturation for 3 min at 98°C; 30 cycles of denaturation for 45 s at 98°C; annealing for 20 s at 62.4°C, and extension for 8 s at 72°C; final extension for 5 min at 72°C. The PCR products were sent to Eurofins GATC Biotech for amplicon sequencing, using the Illlumina MiSeq 2*300 pb with the NGSelect Amplicons 2^nd^ PCR package. The reads were merged by Eurofins. The expected V3 –V5 amplicon size was 569 pb.

### Bioinformatic pipeline

The sequence libraries were trimmed using trimmomatic [36], removing all reads below 500 bp, with a phred quality below 3 for the start and the end of the reads and below an average quality of 10 on a window of 3 base within the reads. FastQC was used to check the quality of the reads before and after the trimming [37]; before to test the trimming parameters, and thereafter to verify that the reads had the necessary quality for the downstream analysis. The following bioinformatic steps were done using QIIME2 2018.11 [38]. Denoising and dereplication of the reads was performed using the denoise and dereplicate single-end sequences (Dada2 denoise-single) with a chimera filtration done using the consensus method [39]. This program is classifying sequences as ASV (amplicon sequence variant) that distinguish sequence variation by a single nucleotide difference [39]. Phylogenetic tree construction was carried by creating a sequence alignment and removing phylogenetically uninformative alignment using MAFFT. Taxonomic affiliation was done using the classify-consensus vsearch program and the TaxAss pipeline [40], which used both a general database, SILVA_132, and a freshwater ecosystem-specific database, FreshTrain [40–42]. Before the taxonomic classification, the dataset was split into two groups using Blastn: sequences with a low percent identity to ecosystem-specific reference sequences, and sequences with high percent identity to ecosystem-specific reference sequences. The group containing the low percent identity sequences was affiliated using the general SILVA_132 database and the group with the high percent identity sequences was affiliated using the Freshtrain database. The two groups were then recombined and used for downstream analysis.

### Biostatistics

All statistical analyses were performed with R software [43] using the package Phyloseq [44], Vegan [45] and EdgeR [46]. Graphical display was done using ggplot2 [47]. ASVs representing more than 9 reads in at least one replicate were kept for downstream analysis. Chloroplast affiliated ASVs were also removed from the dataset. After removal of the low quality, chimera and spurious sequences (false positive sequences) during the bioinformatic treatment and the filtration of the lowest abundant reads, the lowest number of reads in a sample was 18,696 for the first experiment and 17,938 reads for the second experiment. These minimal numbers of reads allowed to have confidence in recovering all the taxa richness present in our samples as indicated by the rarefaction curves (S1 Fig). No rarefaction has been applied on the dataset [48], the data was normalized by relative abundance in percentage by dividing the number of reads affiliated to an ASV by the total read number in the sample and multiply by 100.

Alpha diversity was measured with the richness using the Observed ASV (bacterial richness), evenness with the Pielou index and both Shannon-Wiener and Simpson diversity index [49–51]. The comparison between samples using Beta diversity was done using Weighted Unifrac distance based on the phylogenetic tree build previously under QIIME2 [52] and visualized using Principal Coordinates Analysis (PCoA). For both experiments, Permutational multivariate analysis of variance (PermANOVA) with 999 permutations on the unifrac distance matrices was done to test the significance of the community composition difference between groups [53]. The samples of the first experiment were grouped by volume of filtered water and the second experiment compared the difference two times independently: UD *versus* TF1/3 and UD *versus* VF1/3. The comparison of relative abundance of taxa between the different conditions was done with the package EdgeR [46]. The analysis was performed on the raw reads datasets normalized using the Relative Log Expression method [54]. P-value adjustment was done using the Benjamini-Hochberg procedure [55]. The tested taxonomic ranks were ASV, family/lineage, order, class and phylum. Species and genus taxonomic ranks were not included because the 16S rRNA gene fragment (V3 –V5) did not yielding enough taxonomic depth to be confident in these ranks. Only two conditions were comparable, so the two lower and the two larger filtration volumes were merged for the first experiment as the samples of these two groups clustered together on the PCoA (Fig 3E). For the second experiment, like for the community composition differences test using PermANOVA, the condition VF1/3 and TF1/3 were each tested independents against UD. A False Discovery Rate (FDR) threshold of 0.05 was used to consider a result significant or not.

## Results

### Impact of different filtration volumes on the bacterioplankton community composition

**Fig 2:**
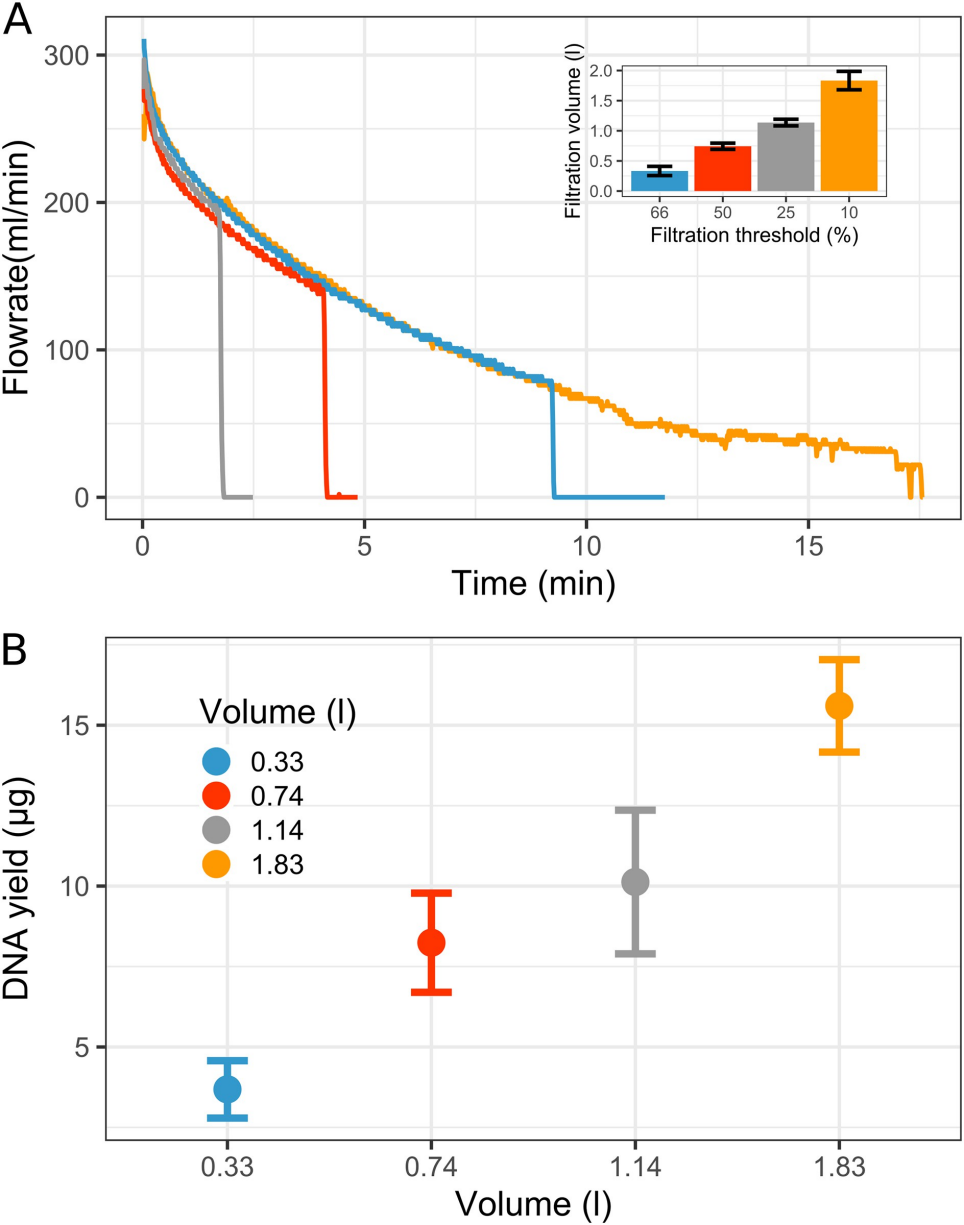
Decrease of flowrate over time during plankton filtrations due to increased biomass loading of the filters, as recorded by the Arduino flowmeter (A), and (B) DNA yields after extraction of the corresponding picoplankton filters. (**A**) The filtrations were stopped at a threshold of 66% (blue), 50% (red), 25% (grey) and 10% (yellow) of the initial, maximal flow rate (F_m_) at the start of the filtration. Each the data for one replicate per triplicate threshold-filtration is shown. (**B**) Average DNA yield (n = 3) in 50 μl extract volume obtained from each set of filters; with standard deviation (SD).

For the first experiment with our flowmeter device (Fig 1), we used a plankton sample taken from Lake Constance (0–20 m integrated sample, sampling site *Wallhausen*) and collected nano- and picoplankton samples by serial filtration through a 180-μm pre-filter onto 5-μm pore size (nanoplankton) and 0.2-μm pore size (picoplankton) polycarbonate membranes; the advantage of polycarbonate was that the material completely dissolved in the phenol-chloroform extraction step during DNA preparation (see Materials and Methods). The filtrations were stopped at four different flowrate-thresholds (F_t_), as monitored with the flowmeter, at F_t_ = 66%, 50%, 25% and 10% of the initial, maximal flow rate (F_m_, 100%). Each threshold filtration was done in triplicate (n = 3). The decrease of flowrate over time is shown in Fig 2A. The total filtration volumes recorded after the filtrations were 0.33 ± 0.07 l, 0.74 ± 0.05 l, 1.14 ± 0.06 l and 1.83 ± 0.15 l, respectively, as illustrated in Fig 2A. The total DNA yields from the picoplankton filters were 3.7 ± 0.9 μg, 8.2 ± 1.5 μg, 10.12 ± 2.2 μg and 15.6 ± 1.4 μg, respectively (Fig 2B). A linear regression (adjusted R-squared = 0.89) confirmed the relation between filtration volume and DNA yield. Notably, the obtained yields also suggested that smaller relative amounts of total DNA per liter of water sample were recovered from the filters if the filters had been loaded with higher amounts of biomass (e.g., 23% less total DNA per liter of water from the 1.83-L vs. the 0.33-L filtration).

We focused on a possible ‘filtration bias’ particularly for the single-cell bacterioplankton community composition, hence, on the picoplankton samples on the 0.2-μm filters [29]. Therefore, amplicon sequencing (Illumina technology) of a 16S rRNA gene fragment (V3-V5 hypervariable region) was applied to evaluated possible differences in the observed bacterioplankton community composition in dependence on the filtration volume. Note that the sequencing data obtained of one of the replicates of the 50% flowrate threshold had to be removed because it was an outlier.

#### Alpha diversity

**Fig 3:**
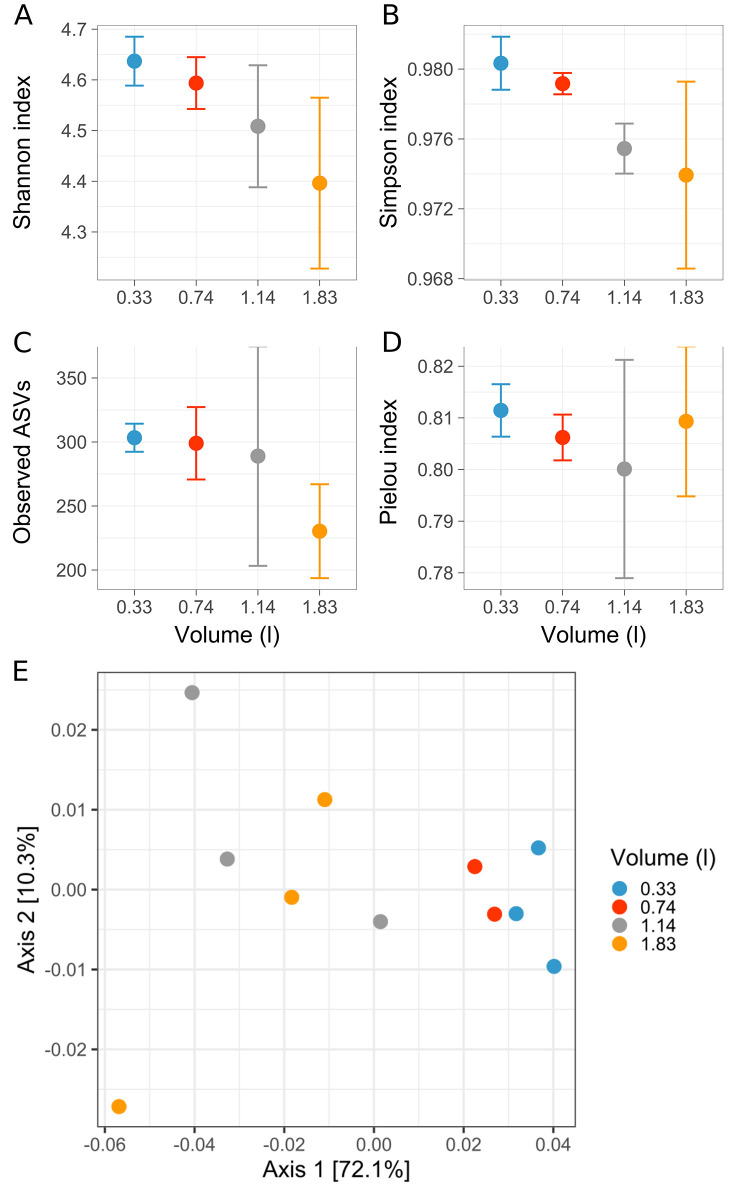
Alpha and beta diversities of the bacterioplankton communities observed for the 0.2-μm filters in dependence on the filtration volume. For the picoplankton filters (0.2-μm pore size) loaded with variable flowrate thresholds (Fig 2A), the bacterioplankton community composition was analyzed by 16S rRNA-gene fragment amplicon sequencing. Alpha diversity is represented by Shannon-Wiener community diversity (**A**), Simpson community diversity (**B**), richness of the observed ASVs (**C**) and Pielou´s community evenness (**D**) indices. Dots represent the average values and the error bar represent the standard deviation of the replicates for each condition (n = 3). (**E**) Principal Coordinate Analysis (PCoA) of the Weighted Unifrac metric calculated from the bacterioplankton community composition.

960 bacterial ASVs were present in the processed dataset after removal of all ASVs that were represented by less than nine reads in at least one replicate. The community diversity was analyzed within each sample (alpha diversity), using the Shannon-Wiener and Simpson indexes. In addition, richness was expressed as the number of observed ASVs and the evenness using the Pielou´s index. Interestingly, increased filtration volume coincided with decreased Shannon and Simpson diversity indices (Fig 3A and 3B). Richness was also decreasing with increased filtration volume, with an average ASV number of 303 for the lowest volume (66% F_m_) and 230 ASVs for the highest volume (10% F_m_) (Fig 3C). Thus, in average 24% of the bacterial taxa detected for the lowest filtration volume were not detectable for the largest filtration volume. The evenness was relatively stable, apart from an increase of variability for increased filtration volumes (Fig 3D). The decrease of diversity observed with the Shannon and Simpson index can be explained by the decreasing richness. This decrease of richness was due to a loss of low-abundant taxa (see explanation below) with increasing filtration volume.

#### Beta diversity

The community structure was analyzed in respect to diversity between samples (beta diversity) and statistically tested for the null hypothesis (H_0_), that the filtration volume had no influence on the observable community composition, and the alternative hypothesis (H_a_), that the filtration volume had an effect on the bacterial community composition. The analyses were done using the Weighted Unifrac distance metric, visualized by Principal Coordinate analysis (PCoA), and the statistical significance was tested using a PermANOVA. The PCoA plot (Fig 3E) illustrates a clear shift of the community compositions in dependence on filtration volume. Each replicate of a filtration threshold clustered together, while the higher-volume conditions showed a higher variability between replicates. The first axis of the PCoA represented 72.1% of the overall variability and the second axis 10.3% (Fig 3E and S2A Fig), which suggested that one variable was the major driver of the observed shift of the community composition, i.e., most likely the variable parameter of our experiment, the filtration volume (biomass loading). This difference of variance may also reflect that an equivalent observable difference between replicates indicate a stronger difference of diversity on the x-axis than the y-axis. PermANOVA results showed a p-value of 0.002 and R^2^ of 0.57. The R^2^ indicated that the filtration volume explained 57% of the community composition variability that, coupled with the p-value <0.05, allowed to reject H_0_. Under these conditions, we can conclude that the filtration volume indeed had an impact on the observed microbial community composition.

#### Individual taxonomic groups affected by filtration volume

**Fig 4:**
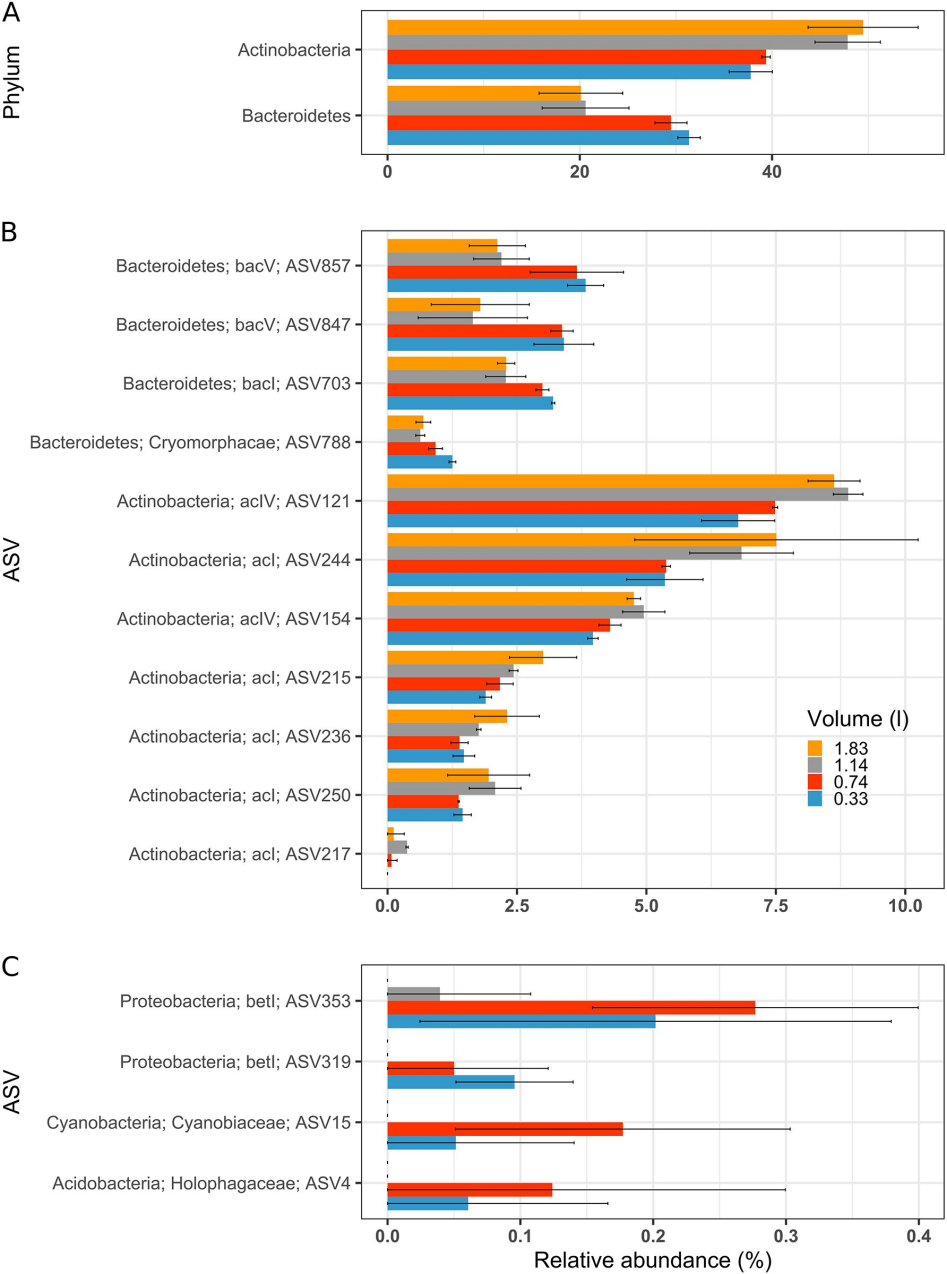
Representative taxa that displayed significant changes in relative abundances in dependence on the filtration volumes used. Shown are (**A**) phyla-taxonomic rank, (**B**) and (**C**) as ASV with family and phyla affiliation. The relative abundance is expressed as percent (%) reads for a specific taxon relative to all reads observed. Error bars represent the standard deviation (n = 3). Note the different x scales.

Next, we examined which taxonomic groups were specifically affected by the filtration volume, using EdgeR to compare the relative abundance changes between conditions. Only two conditions can be compared so the data of the two lowest (0.33 and 0.74 L) and the two highest (1.14 and 1.83 L) filtration volumes were grouped together as their replicates clustered closely on the PCoA, indicating similar community composition (Fig 3E). All taxonomic ranks were analyzed with the exception of genus and species which were rarely assigned in our dataset. The results showed that the community composition was affected at all taxonomic levels by the filtration volume, in that two phyla, six classes, eight orders, 14 families and 15 ASVs showed a significant difference in relative abundance, as illustrated in Fig 4A, 4B and S3A–S3C Fig. The number of affected taxa is minor compared to the total dataset, as they represented 11.8%, 17.1%, 9.8%, 9.0% and 1.6% of the total phylum, class, order, family/lineage and ASV, but represented a majority of the relative abundance as these few taxa represented between 34.0% (ASV) to 78.8% (class) of the total abundance.

Most different taxa in relative abundance, in dependence on filtration volume were affiliated to Actinobacteria and Bacteroidetes, together representing 69% of all affected taxa. The differences followed opposing trends, with Actinobacteria increasing with increased filtration volume (up to 38% increase at the phylum rank) and Bacteroidetes decreasing with increased filtration volume (up to 29% decrease for the phylum rank) (Fig 4A). The percentage of relative abundance change varied from a maximal increase of 67% for the family/lineage Sporichthyaceae (S3A Fig) to a maximal decrease of 50% for ASV847 affiliated to the family/lineage bacV (Fig 4A, S1 Table). Four others ASVs not affiliated with either Actinobacteria or Bacteroidetes were only detected for the lowest filtration volumes (0.33 and 0.74 L) (Fig 4C). These four ASVs were detected by EdgeR because their initial relative abundance was high enough in the low filtration volumes, yet we can expect that more ASVs may display the same pattern. This decrease of total number of ASV detected in the highest volumes could explain why the richness was decreasing with increased filtration volume (Fig 3C).

### Normalization of the plankton filtration bias using our flowmeter device

With the next experiment, we tested whether our flowmeter device may be helpful to normalize the filtration bias described above, by normalizing biomass loading of the filters using threshold filtration, for the bacterioplankton community as represented on the 0.2-μm filters. One of the replicates of condition VF1/3 (see below) had to be removed because it was an outlier, leaving three replicate for this condition.

**Fig 5:**
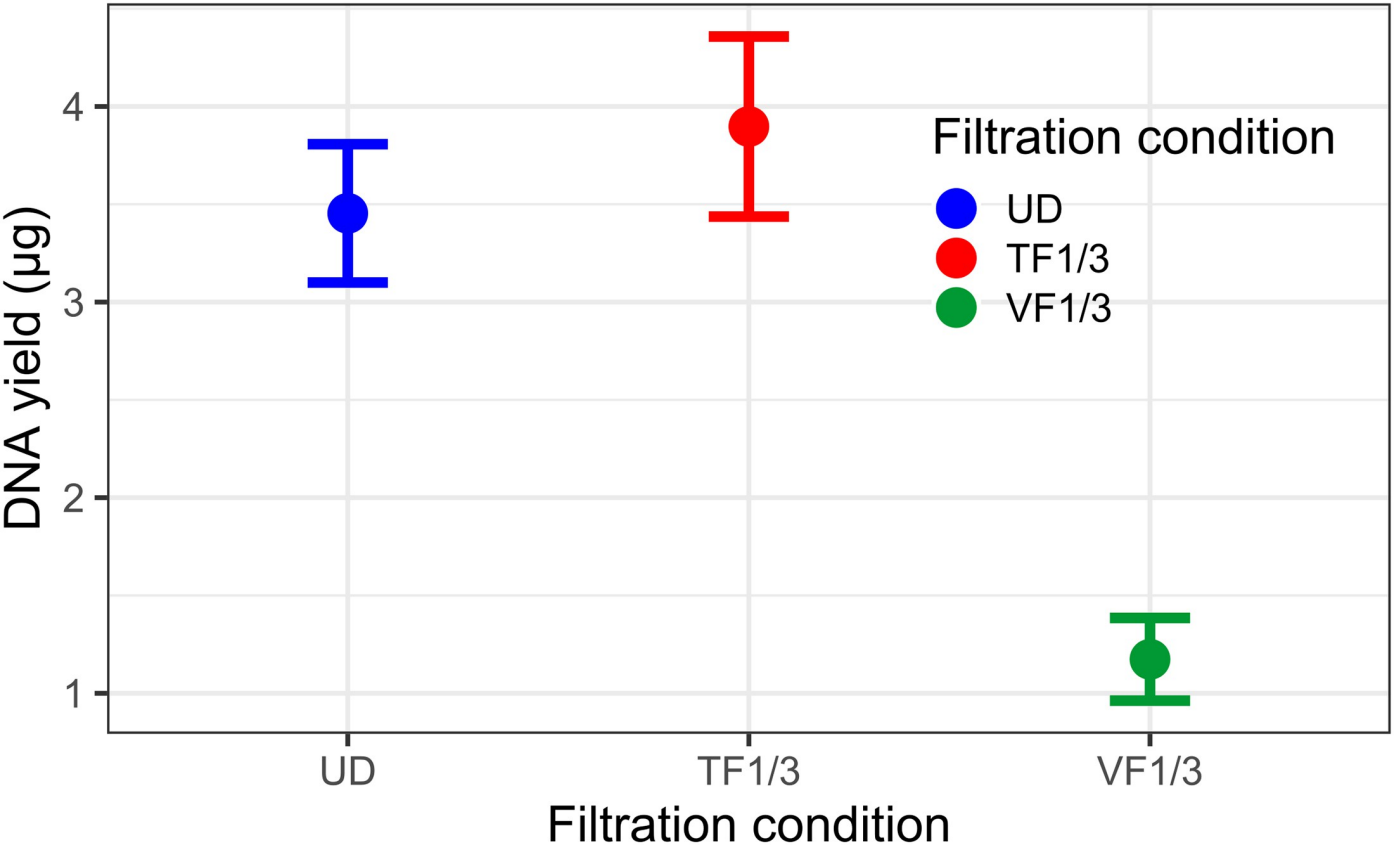
DNA yield after extraction of the picoplankton filters. (**A**) UD corresponds to the undiluted water condition, TF1/3 to the filtration of the 3-fold diluted water sample using threshold filtration with the flowmeter device, and VF1/3 to the filtration of the 3-fold diluted water using the same filtration volume as for the undiluted condition.

Another Lake Constance sample (0–20 m depth, *Wallhausen*) was filtered at its given plankton density (termed ‘undiluted condition’, UD) and after the sample had been diluted three-fold (1:3), each with threshold filtrations (TF) using the flowmeter device and stopping the filtrations again at Ft = 50% F_m_. The total filtration volumes recorded for the undiluted sample were 0.57 ± 0.06 l (n = 8) and for the 1:3-diluted sample (termed TF1/3) 1.71 ± 0.04 l (n = 4), hence, the 3-fold volume. As a third filtration condition (termed ‘fixed-volume filtration’ VF1/3), we filtered the three-fold diluted sample with the same volume as the undiluted sample (0.57 L), hence without the flowmeter device and as control of the filtration bias (n = 3). The DNA yields obtained for the UD and TF1/3 conditions were relatively similar with 3.5 ± 0.4 μg and 3.9 ± 0.5 μg, while the VF1/3 condition DNA yields were about 3-fold lower with 1.2 ± 0.2 μg, as expected (Fig 5).

#### Alpha and beta diversities

**Fig 6:**
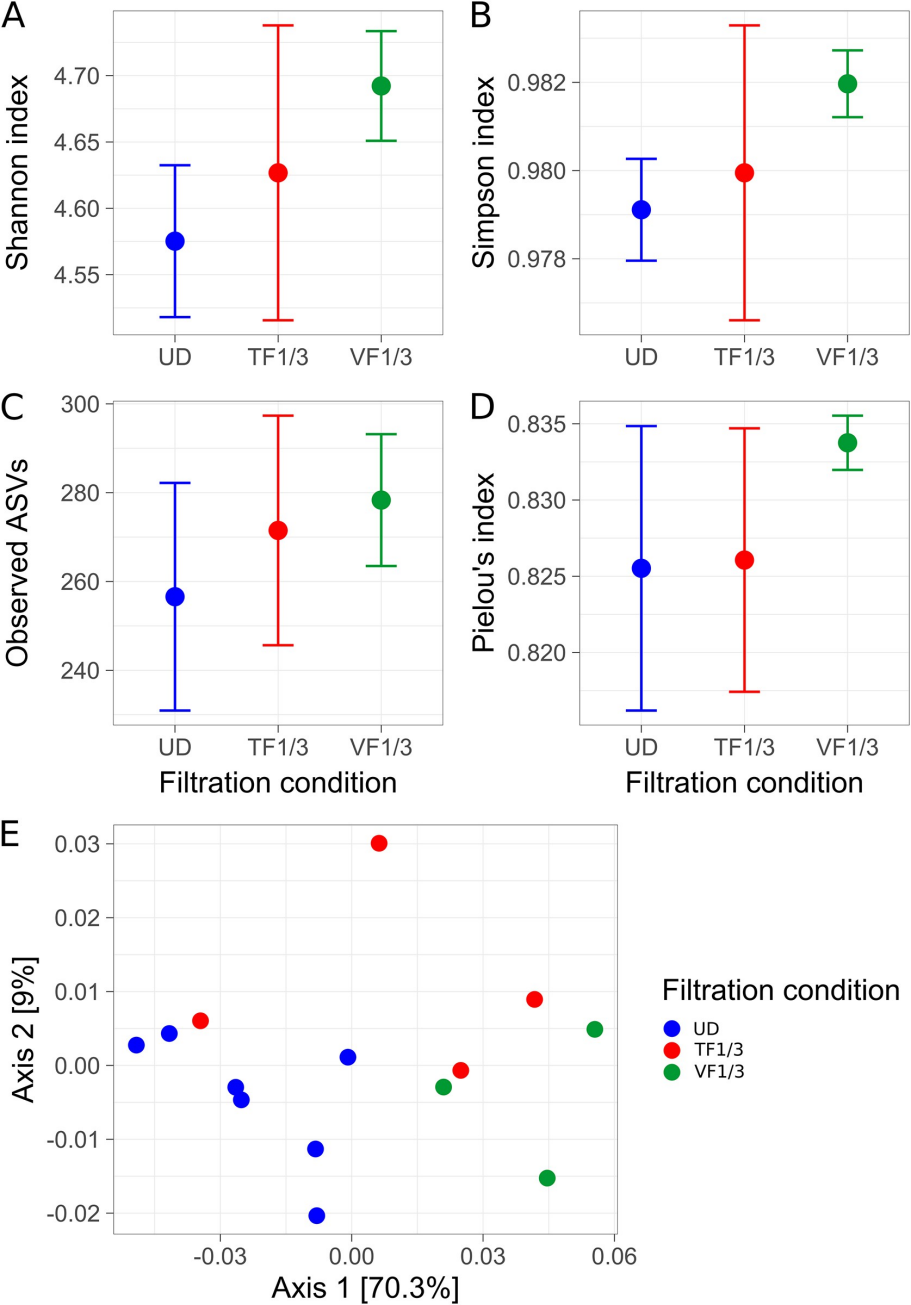
Alpha and beta diversities of undiluted and diluted bacterioplankton collected on 0.2-μm filters by threshold filtration in comparison to fixed-volume filtration. A Lake Constance water sample was filtered undiluted (UD) and 3-fold diluted (threshold filtration, TF1/3) using each the flowmeter device at 50% flow-rate threshold. For control, the 3-fold diluted sample was also filtered with the same volume as the undiluted sample (fixed-volume filtration, VF1/3). Alpha diversity is represented by Shannon-Wiener community diversity (**A**), Simpson community diversity (**B**), richness of the observed ASVs (**C**) and Pielou´s community evenness (**D**) indices. Dots represent the average values and the error bar represent the standard deviation of the replicates for each condition. (**E**) Principal Coordinate Analysis (PCoA) of the Weighted Unifrac metric calculated from the bacterioplankton community composition.

The Shannon-Wiener and Simpson values of the UD condition were smaller than the VF1/3 condition (non-overlapping standard deviations [SD]), while the TF1/3 diversity values were in between the two conditions (SD overlapping with both UD and VF1/3 conditions) (Fig 6A and 6B). The average number of ASVs in the processed data was 257 ± 27, 271 ± 26 and 278 ± 15 for the conditions UD, TF1/3 and VF1/3 respectively, hence the richness in the diluted samples seemed to be slightly higher, although remaining relatively close to each other (e.g., visible by all three standard deviations overlapping) (Fig 6C). The same trend was observed for the evenness (Pielou), while the standard deviation (SD) for VF1/3 was much smaller (Fig 6D).

For the beta diversity, the first axis of the PCoA represented 69.3% of the variability and the second axis 8.4% (Fig 6E and S2B Fig). Hence, one factor was the major driver of the observed variability, like in the first experiment. An equivalent difference between replicates indicates a stronger difference of diversity on the x-axis than the y-axis. Replicates of each condition clustered together, with the VF1/3 filtrations more clearly separated from the undiluted filtrations, and the TF1/3 filtrations in between (Fig 6E).

The goal of this experiment was to examine whether the filtration bias may be normalized when filtrations are done using the flowrate device (undiluted *vs*. TF1/3) in comparison to fixed-volume filtration (undiluted *vs*. VF1/3). Hence, the statistical hypotheses for the analysis of the fixed-volume condition were, (H_0_) plankton density does not affect the observed diversity (centroid of the compared group are equivalent), and (H_a_) plankton density does affect the observed diversity (centroid of the compared group are not equivalent). The PermANOVA output was a p-value of 0.027 and a R^2^ of 0.66, thus, we can reject H_0_ and conclude that the plankton density (filter loading) impacted the diversity, when using fixed-volume filtration. For the threshold filtration, which may have no impact (H_0_) or may have an impact (H_a_) on the diversity in the diluted sample, the p-value was 0.114 and the R^2^ 0.20, and therefore, we cannot reject H_0_. Hence, at the community level, a difference in plankton density in the diluted sample did not statistically affect the observed community when using our flowrate device, suggesting that threshold filtrations may compensate for the filtration bias.

#### Individual taxonomic groups affected

**Fig 7:**
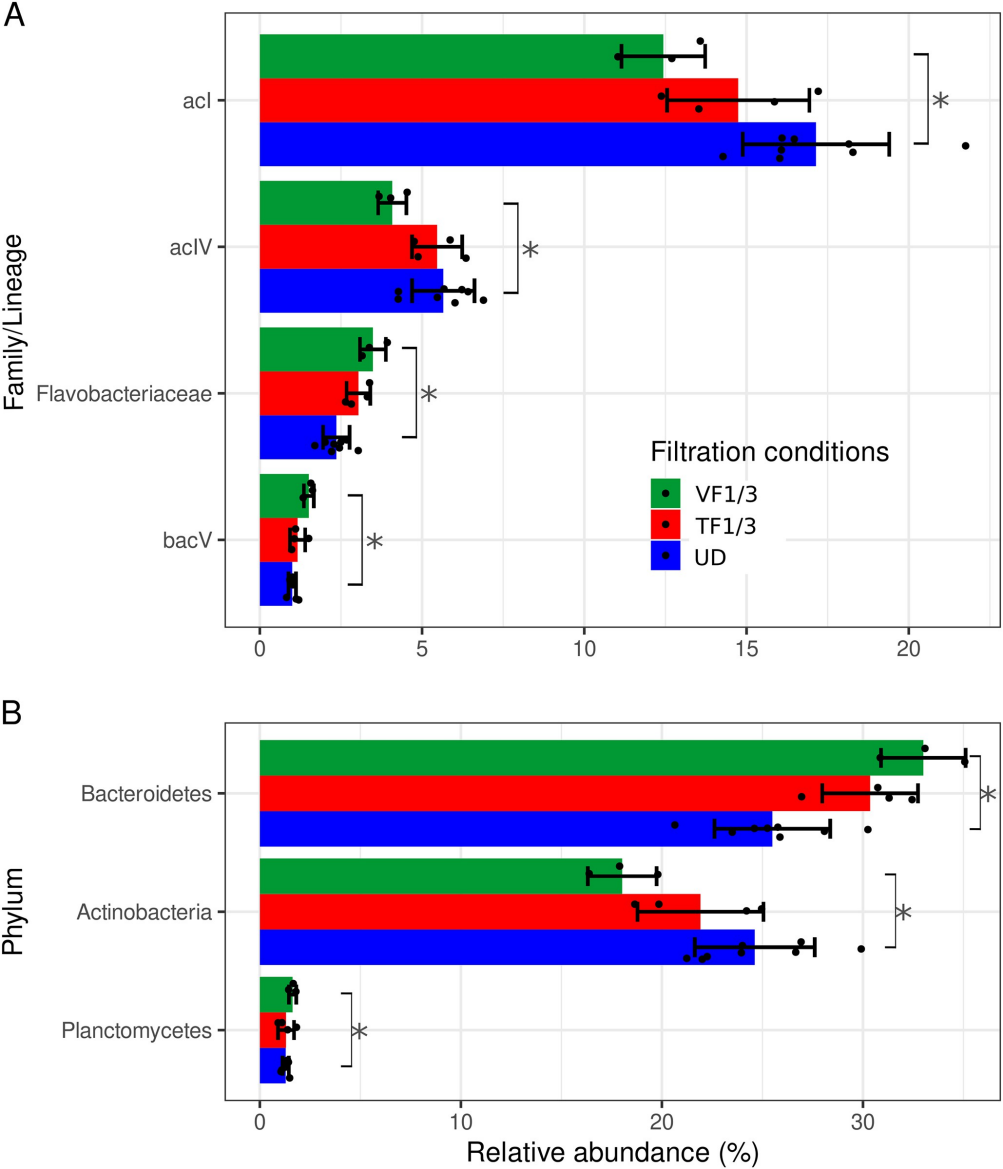
Specific taxa that displayed different relative abundances in dependence on the filtration method used. Lake Constance water was filtered undiluted and 1:3 diluted (threshold filtration, TF1/3) using the flowmeter device, and the 1:3-diluted sample was also filtered with the same volume as the undiluted sample (fixed-volume filtration, VF1/3). Shown are examples on the (A) family and (B) phyla level. Error bars represent the standard deviation (n = 3). Significance is flagged with one star (*). Note the variation of y axis scales.

EdgeR was used to compare the relative abundance change of taxa between the conditions (genus and species excluded). For the undiluted *vs*. VF1/3 condition and the confirmation of the filtration bias, all taxonomic ranks except order showed taxa with abundance significantly changed, i.e., three phyla, four classes, four families/lineages and two ASVs (Fig 7 and S4 Fig). Like in the first experiment, the number of affected taxa is minor compared to the total number of taxa as they represented 18.8%, 12.9%, 2.5% and 0.4% of the total taxa in their respective taxonomic rank. The relative abundance of the impacted taxa is less dominating, representing between 2.4% (ASV) to 51.6% (class) of the total relative abundance. As with the first experiment, most taxa belonged to the phyla Actinobacteria and Bacteroidetes and these two phyla showed a decrease of 27% and an increase of 30% of relative abundance respectively, when the diluted plankton had been filtered by fixed-volume filtration (Fig 7B). The decrease in relative abundance in relation to plankton dilution was up to 34% for the family/lineage acI (S4 Fig and S2 Table). In addition, significant increase of also phylum Planktomycetes (28%) and its belonging class Phycisphaerae (40%) (Fig 7A) was detected for the VF1/3 condition in this experiment.

The undiluted *vs*. TF1/3 condition showed no taxa, whichever taxonomic rank, that were changed at statistically significant level. This is illustrated in Fig 7 and S4 Fig, where the standard deviations for the TF1/3 condition are always overlapping with these of the undiluted condition, hence, showing a difference not strong enough to be significant, while the SD for the VF1/3 condition illustrate significant differences (filtration bias).

## Discussion

The first experiment using the flowmeter device confirmed and expanded on the description of a pico(bacterio)plankton filtration bias reported previously [29, 56]. Less bacterial taxa could be detected for the largest sample volume filtered (Fig 3A), which may appear counterintuitive, as more water was filtered, so more bacterioplankton biomass was collected, which should lead to a better detection of also the lowest-abundant taxa. As described in the Introduction, we considered whether the cause of such a volume-dependent filtration bias may be a build-up of a filtration cake during serial filtration, which is increasingly acting as an additional, smaller-pore size filter, thereby increasingly retaining microorganisms on this filter that would normally belong into the filtrate and thus onto the next smaller pore-size filter. Through the implied biomass dependence, samples with different plankton densities may produce a similar bias if each the same volume of water is filtered, as confirmed with the second experiment (Fig 6; UD *vs*. VF1/3 conditions). This effect is particularly relevant for plankton diversity studies in lakes of temperate regions, where the plankton density can change drastically in between winter mixing and the vegetative season, for example for deep Lake Constance, which is the subject of our own plankton diversity studies. Lake Constance currently displays Secchi depths in winter of down to 21 m (February 2022, *Überlinger* See) and up to 2.5 m during spring bloom and summer.

Members of the phyla Actinobacteria and Bacteroidetes were among the taxa most affected by increased filtration volume. Typical freshwater Bacteroidetes decreased in relative abundance for the 0.2-μm filters (Fig 4), as well as similarly affected family Pedosphaeracae, and phyla Verrucomicrobia, Holophagaceae and Acidobacteria (S3 Fig), which are known to be filamentous or particle-associated bacteria [57, 58]. They may increasingly be captured by the biomass cake on the 5-μm filter and therefore be detected at lower relative abundance on the 0.2-μm filters with increasing filtration volume. *Vice versa*, a major part of the typical freshwater Actinobacteria are contributed by very small, ‘ultramicrobacteria’ [41, 59, 60], and an increased biomass cakes on the filters may have retained these cells more efficiently on the 0.2-μm filter for higher filtration volumes.

The 16S rDNA amplicon sequencing as performed in this study, was likely also subject of a ‘DNA-template dilution bias’ for the PCR [11]. In the study by Wu *et al*., it was reported that the number of taxa detected was decreasing with higher template dilution (i.e., decreasing probability for low abundant taxa-DNA to be amplified), while the relative abundance of the detected ASVs was not significantly changed by the different template dilution [11]. In our study, the different DNA yields recovered from the different filtration volumes (Fig 2B) led us to apply higher template dilution for the DNA extracts of the highest filtration volume, in order to obtain uniform PCR conditions. Thus, this additional bias may also be a factor that contributed to the observed decrease of richness through loss of some of the lowest-abundant taxa. The lowest filtration volume showed 93 ASVs more than the highest, and these bacterial taxa belong to the very-low abundant bacteria, with an average relative abundance of 0.029%. For the first experiment, four ASVs may illustrate this DNA-template dilution bias, as they were detectable for the low filtration volume and not anymore for the high filtration volume (Fig 4C). However, the design of our experiments does not allow to clearly identify which bias, DNA-template dilution bias or filtration bias, was responsible for the loss of abundance observed in this study. Nevertheless, the difference in relative ASV abundance as observed with the different filtration volumes is due to the filtration bias, as the DNA-template dilution bias only affects the ability to detect ASVs.

In the second experiment, no statistically significant differences were observed between the undiluted and diluted plankton when collected by flowrate-threshold filtration (Fig 6). The 50%-threshold filtration allowed for the collection of equivalent amounts of DNA regardless of the three-fold dilution of the original water sample (Fig 5), and thus, a different dilution of the DNA for PCR amplification was not necessary, which likely eliminated also a DNA-template dilution bias for the UD and TF1/3 samples [11]. However, the number of taxa for the VF1/3 condition was slightly higher (Fig 6A), and this increase may indeed be due to DNA-template dilution bias, as the UD and TF1/3 DNA samples had to be diluted three-fold relative to the VF1/3 DNA sample for PCR. Hence, we are tempted to speculate whether the compensation of the filtration bias by collecting equivalent amount of biomass, resulting in similar dilution for the PCR, may also compensate for the DNA-template dilution bias.

**Fig 8:**
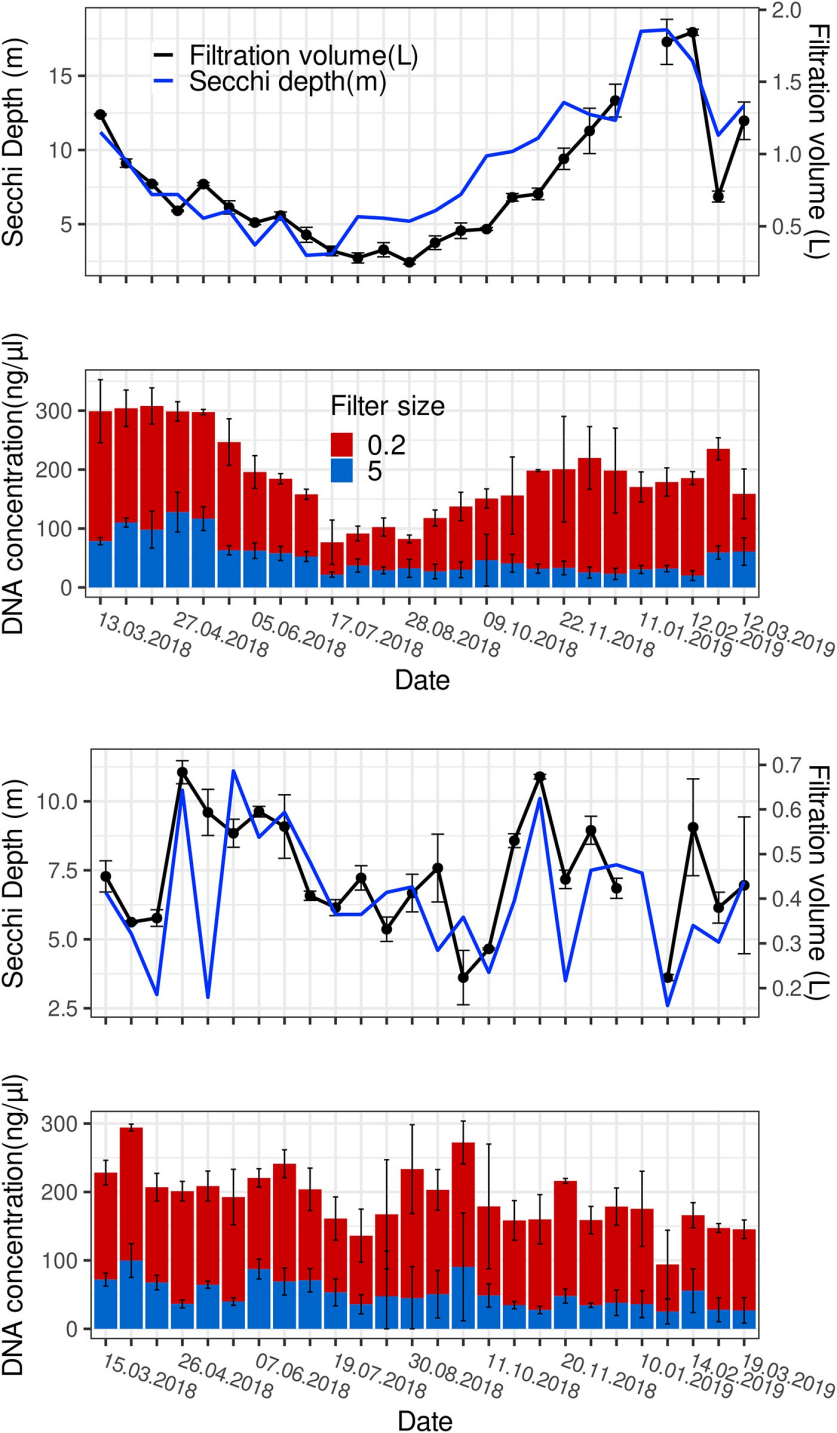
Secchi depths, filtration volumes recorded for threshold filtrations, and DNA yields obtained from seasonal sampling of Upper and Lower Lake Constance plankton. The sampling was done between March 2018 –March 2019 at Upper Lake Constance, *Überlinger See* (A, B), and Lower Lake Constance, *Zeller See* (C, D). Integrated samples from 0–20 m water depth were collected and filtered using the flowmeter device set to a threshold of 50% Fm. The filtration volumes and DNA yields are shown as average of triplicates per sampling date; error bars represent standard deviation. Note that the DNA concentrations from extracting both size-class filters, the 5.0-μm (red) and 0.2-μm (blue) pore-size filters, are shown.

We considered whether this method of threshold filtration may be useful for sampling across depth profiles and along seasonal cycles of temperate lakes, and we therefore tested our filtration setup (Fig 1) (at 50% threshold) in a yearly sampling campaign for Lake Constance, when collecting every two weeks a sample integrated over 0–20 m water depth as a representation of the photic zones in two different lake parts, Upper Lake (*Überlinger See*) and Lower Lake Constance (*Zeller See*). In Fig 8, the Secchi depth as a representation of plankton density is shown, and the recorded sample volumes at which the 50%-threshold was reached, and the DNA yields obtained from extraction of the 5-μm and 0.2-μm pore size filters. Increased Secchi depths correlated well with increased filtration volumes in order to reach the 50%-threshold. For example, a sample taken at >16 m Secchi depth required almost 2 l water, and at 5 m Secchi depth around 0.4–0.7 l, while similar amounts of DNA were recovered from the filters at both high and low Secchi depths, particularly throughout the seasonal cycle of the *Zeller See* (Fig 8C and 8D). For *Überlinger See*, the DNA yield from threshold filtration tended to be highest at low Secchi depth in winter and during the spring bloom (Fig 7A and 7B), while in summer and beginning of fall, the DNA yields were unexpectedly low. We believe that this may have resulted from calcite precipitation, typical for Upper Lake Constance in summer [61–63], in that additional calcite particles most likely led to a faster clogging of the filters, thus to a lower ratio of cellular biomass collected until the 50%-threshold was reached, and hence to lower amounts of DNA extracted. Hence, it appeared that the effect of threshold filtration for normalizing plankton DNA yields may itself be distorted by calcite (or other inorganic) particles contributing to the clogging of the filters, and that this needs to be considered for any future application of the device.

In conclusion, we demonstrated a filtration bias in aquatic picoplankton (single-cell bacterioplankton) diversity studies if size-class filtration, DNA extraction, PCR and sequencing of phylogenetic markers is applied. We also presented a solution to resolve this bias, by employing flowrate-threshold filtration. This new filtration technique can be useful to eliminate bias in plankton diversity studies that use PCR-amplicon sequencing for estimating relative abundances of taxa. It can also be useful for estimating absolute abundances of taxa by quantitative PCR (qPCR) and Droplet Digital PCR (ddPCR) since the Arduino devices (Fig 1 and S5 Fig) also record the total filtration volume for each threshold filtration, for calculating template DNA concentration relative to sample volume. The plankton filtration bias can be expected to affect not only DNA-based community analyses. Transcriptomic and proteomic analyses also require collection of plankton biomass by filtration, making these methodological pipelines also susceptible. This may particularly be relevant for multi-omics analyses if different plankton samples for each pipeline are collected by different filtration methods and/or volumes, e.g., for determining the plankton metagenome and its transcriptional and/or translational activity. The relevance of the filtration bias may also expand to comparisons of freshwater and marine water bodies and mesocosm experiments in which plankton densities vary strongly, and to samples collected at different depths in lakes or other water bodies with several biomass (chorophyll-a and deep water) maxima and minima. Hence, we believe that using flowrate-threshold filtration and a normalization of the biomass yield for each plankton filter can increase the accuracy of also such analyses.

## Supporting information

S1 Fig Rarefaction curves of the samples sequenced for the first (A) and second filtration experiment (B) as described in the main text. (TIF)

S2 Fig Eigenvalues of all Principal Coordinate Analysis axes for the first (A) and second filtration experiment (B) as described in the main text. (TIF)

S3 Fig Representative taxa that displayed significant changes in relative abundances for the first filtration experiment described in the main text, on the (A) family/lineage, (B) order and (C) class rank. (TIF)

S4 Fig Representative taxa that displayed significant changes in relative abundances for the second filtration experiment described in the main text, on the (A) ASV (B) class rank. (TIF)

S5 Fig Photographic illustration of a professionalized flowmeter device. (TIF)

S1 Table Taxa with relative abundances significantly changed by the filtration bias as introduce by the different filtration volumes (first experiment) and as identified using EdgeR. (XLSX)

S2 Table Taxa with relative abundances significantly changed for the VF1/3-condition compared to UD condition in the second experiment and identified using EdgeR. (XLSX)

## References

[1] Pierrou U. The Global Phosphorus Cycle. Ecological Bulletins. 1976; 75–88.

[2] Jones RI. Phytoplankton, Primary Production and Nutrient Cycling. In: Hessen DO, Tranvik LJ, editors. Aquatic Humic Substances: Ecology and Biogeochemistry. Berlin, Heidelberg: Springer; 1998. pp. 145–175.

[3] Jetten MSM. The microbial nitrogen cycle. Environmental Microbiology. 2008;10: 2903–2909. doi: 10.1111/j.1462-2920.2008.01786.x 18973618

[4] Madsen EL. Microorganisms and their roles in fundamental biogeochemical cycles. Current Opinion in Biotechnology. 2011;22: 456–464. doi: 10.1016/j.copbio.2011.01.008 21333523

[5] Hugoni M, Taib N, Debroas D, Domaizon I, Jouan Dufournel I, Bronner G, et al. Structure of the rare archaeal biosphere and seasonal dynamics of active ecotypes in surface coastal waters. Proc Natl Acad Sci U S A. 2013;110: 6004–6009. doi: 10.1073/pnas.1216863110 23536290 PMC3625260

[6] Hugoni M, Vellet A, Debroas D. Unique and highly variable bacterial communities inhabiting the surface microlayer of an oligotrophic lake. Aquatic Microbial Ecology. 2017;79: 115–125. doi: 10.3354/ame01825

[7] Chafee M, Fernàndez-Guerra A, Buttigieg PL, Gerdts G, Eren AM, Teeling H, et al. Recurrent patterns of microdiversity in a temperate coastal marine environment. ISME J. 2018;12: 237–252. doi: 10.1038/ismej.2017.165 29064479 PMC5739018

[8] Delhomme N, Sundström G, Zamani N, Lantz H, Lin Y-C, Hvidsten TR, et al. Serendipitous Meta-Transcriptomics: The Fungal Community of Norway Spruce (Picea abies). PLOS ONE. 2015;10: e0139080. doi: 10.1371/journal.pone.0139080 26413905 PMC4586145

[9] Grassl N, Kulak NA, Pichler G, Geyer PE, Jung J, Schubert S, et al. Ultra-deep and quantitative saliva proteome reveals dynamics of the oral microbiome. Genome Med. 2016;8. doi: 10.1186/s13073-016-0293-0 27102203 PMC4841045

[10] Krakat N, Anjum R, Demirel B, Schröder P. Methodological flaws introduce strong bias into molecular analysis of microbial populations. J Appl Microbiol. 2017;122: 364–377. doi: 10.1111/jam.13365 27914209

[11] Wu J-Y, Jiang X-T, Jiang Y-X, Lu S-Y, Zou F, Zhou H-W. Effects of polymerase, template dilution and cycle number on PCR based 16 S rRNA diversity analysis using the deep sequencing method. BMC Microbiology. 2010;10: 255. doi: 10.1186/1471-2180-10-255 20937143 PMC2964677

[12] Berry D, Mahfoudh KB, Wagner M, Loy A. Barcoded Primers Used in Multiplex Amplicon Pyrosequencing Bias Amplification. Appl Environ Microbiol. 2011;77: 7846–7849. doi: 10.1128/AEM.05220-11 21890669 PMC3209180

[13] van Dijk EL, Jaszczyszyn Y, Thermes C. Library preparation methods for next-generation sequencing: Tone down the bias. Experimental Cell Research. 2014;322: 12–20. doi: 10.1016/j.yexcr.2014.01.008 24440557

[14] Filker S, Sommaruga R, Vila I, Stoeck T. Microbial eukaryote plankton communities of high-mountain lakes from three continents exhibit strong biogeographic patterns. Mol Ecol. 2016;25: 2286–2301. doi: 10.1111/mec.13633 27029537 PMC4976798

[15] Llorens-Marès T, Triadó-Margarit X, Borrego CM, Dupont CL, Casamayor EO. High Bacterial Diversity and Phylogenetic Novelty in Dark Euxinic Freshwaters Analyzed by 16S Tag Community Profiling. Microb Ecol. 2016;71: 566–574. doi: 10.1007/s00248-015-0696-2 26552395

[16] Díez B, Pedrós-Alió C, Massana R. Study of genetic diversity of eukaryotic picoplankton in different oceanic regions by small-subunit rRNA gene cloning and sequencing. Appl Environ Microbiol. 2001;67: 2932–2941. doi: 10.1128/AEM.67.7.2932-2941.2001 11425705 PMC92964

[17] Allen LZ, Allen EE, Badger JH, McCrow JP, Paulsen IT, Elbourne LD, et al. Influence of nutrients and currents on the genomic composition of microbes across an upwelling mosaic. The ISME Journal. 2012;6: 1403–1414. doi: 10.1038/ismej.2011.201 22278668 PMC3379637

[18] Fuchsman CA, Staley JT, Oakley BB, Kirkpatrick JB, Murray JW. Free-living and aggregate-associated Planctomycetes in the Black Sea. FEMS Microbiol Ecol. 2012;80: 402–416. doi: 10.1111/j.1574-6941.2012.01306.x 22251018

[19] Bradford TM, Morgan MJ, Lorenz Z, Hartley DM, Hardy CM, Oliver RL. Microeukaryote community composition assessed by pyrosequencing is associated with light availability and phytoplankton primary production along a lowland river. Freshwater Biology. 2013;58: 2401–2413. doi: 10.1111/fwb.12219

[20] Hugoni M, Etien S, Bourges A, Lepère C, Domaizon I, Mallet C, et al. Dynamics of ammonia-oxidizing Archaea and Bacteria in contrasted freshwater ecosystems. Res Microbiol. 2013;164: 360–370. doi: 10.1016/j.resmic.2013.01.004 23395876

[21] Eiler A, Heinrich F, Bertilsson S. Coherent dynamics and association networks among lake bacterioplankton taxa. ISME J. 2012;6: 330–342. doi: 10.1038/ismej.2011.113 21881616 PMC3260505

[22] Baltar F, Palovaara J, Unrein F, Catala P, Horňák K, Šimek K, et al. Marine bacterial community structure resilience to changes in protist predation under phytoplankton bloom conditions. ISME J. 2016;10: 568–581. doi: 10.1038/ismej.2015.135 26262814 PMC4817682

[23] Taguchi S, Laws EA. On the microparticles which pass through glass fiber filter type GF/F in coastal and open waters. J Plankton Res. 1988;10: 999–1008. doi: 10.1093/plankt/10.5.999

[24] Knefelkamp B, Carstens K, Wiltshire KH. Comparison of different filter types on chlorophyll-a retention and nutrient measurements. Journal of Experimental Marine Biology and Ecology. 2007;345: 61–70. doi: 10.1016/j.jembe.2007.01.008

[25] Eiler A, Bertilsson S. Composition of freshwater bacterial communities associated with cyanobacterial blooms in four Swedish lakes. Environ Microbiol. 2004;6: 1228–1243. doi: 10.1111/j.1462-2920.2004.00657.x 15560821

[26] Frias-Lopez J, Shi Y, Tyson GW, Coleman ML, Schuster SC, Chisholm SW, et al. Microbial community gene expression in ocean surface waters. Proc Natl Acad Sci USA. 2008;105: 3805–3810. doi: 10.1073/pnas.0708897105 18316740 PMC2268829

[27] Hunt DE, Lin Y, Church MJ, Karl DM, Tringe SG, Izzo LK, et al. Relationship between Abundance and Specific Activity of Bacterioplankton in Open Ocean Surface Waters. Appl Environ Microbiol. 2013;79: 177–184. doi: 10.1128/AEM.02155-12 23087033 PMC3536108

[28] Lee S, Kang Y-C, Fuhrman JA. Imperfect retention of natural bacterioplankton cells by glass fiber filters. Marine Ecology Progress Series. 1995;119: 285–290.

[29] Padilla CC, Ganesh S, Gantt S, Huhman A, Parris DJ, Sarode N, et al. Standard filtration practices may significantly distort planktonic microbial diversity estimates. Front Microbiol. 2015;6. doi: 10.3389/fmicb.2015.00547 26082766 PMC4451414

[30] Rusch DB, Halpern AL, Sutton G, Heidelberg KB, Williamson S, Yooseph S, et al. The Sorcerer II Global Ocean Sampling expedition: northwest Atlantic through eastern tropical Pacific. PLoS Biol. 2007;5: e77. doi: 10.1371/journal.pbio.0050077 17355176 PMC1821060

[31] Huttenhower C, Gevers D, Knight R, Abubucker S, Badger JH, Chinwalla AT, et al. Structure, function and diversity of the healthy human microbiome. Nature. 2012;486: 207–214. doi: 10.1038/nature11234 22699609 PMC3564958

[32] Boers SA, Hays JP, Jansen R. Micelle PCR reduces chimera formation in 16S rRNA profiling of complex microbial DNA mixtures. Sci Rep. 2015;5: 14181. doi: 10.1038/srep14181 26373611 PMC4570986

[33] Schuurman T, de Boer RF, Kooistra-Smid AMD, van Zwet AA. Prospective Study of Use of PCR Amplification and Sequencing of 16S Ribosomal DNA from Cerebrospinal Fluid for Diagnosis of Bacterial Meningitis in a Clinical Setting. J Clin Microbiol. 2004;42: 734–740. doi: 10.1128/JCM.42.2.734-740.2004 14766845 PMC344470

[34] Parada AE, Needham DM, Fuhrman JA. Every base matters: assessing small subunit rRNA primers for marine microbiomes with mock communities, time series and global field samples. Environmental Microbiology. 2016;18: 1403–1414. doi: 10.1111/1462-2920.13023 26271760

[35] Walters W, Hyde ER, Berg-Lyons D, Ackermann G, Humphrey G, Parada A, et al. Improved Bacterial 16S rRNA Gene (V4 and V4-5) and Fungal Internal Transcribed Spacer Marker Gene Primers for Microbial Community Surveys. mSystems. 2016;1. doi: 10.1128/mSystems.00009-15 27822518 PMC5069754

[36] Bolger AM, Lohse M, Usadel B. Trimmomatic: a flexible trimmer for Illumina sequence data. Bioinformatics. 2014;30: 2114–2120. doi: 10.1093/bioinformatics/btu170 24695404 PMC4103590

[37] Simon A. Babraham Bioinformatics—FastQC A Quality Control tool for High Throughput Sequence Data. 2010 [cited 10 May 2020]. https://www.bioinformatics.babraham.ac.uk/projects/fastqc/

[38] Bolyen E, Rideout JR, Dillon MR, Bokulich NA, Abnet CC, Al-Ghalith GA, et al. Reproducible, interactive, scalable and extensible microbiome data science using QIIME 2. Nat Biotechnol. 2019;37: 852–857. doi: 10.1038/s41587-019-0209-9 31341288 PMC7015180

[39] Callahan BJ, McMurdie PJ, Rosen MJ, Han AW, Johnson AJA, Holmes SP. DADA2: High-resolution sample inference from Illumina amplicon data. Nature Methods. 2016;13: 581–583. doi: 10.1038/nmeth.3869 27214047 PMC4927377

[40] Rohwer RR, Hamilton JJ, Newton RJ, McMahon KD. TaxAss: Leveraging a Custom Freshwater Database Achieves Fine-Scale Taxonomic Resolution. mSphere. 2018;3. doi: 10.1128/mSphere.00327-18 30185512 PMC6126143

[41] Newton RJ, Jones SE, Eiler A, McMahon KD, Bertilsson S. A guide to the natural history of freshwater lake bacteria. Microbiol Mol Biol Rev. 2011;75: 14–49. doi: 10.1128/MMBR.00028-10 21372319 PMC3063352

[42] Quast C, Pruesse E, Yilmaz P, Gerken J, Schweer T, Yarza P, et al. The SILVA ribosomal RNA gene database project: improved data processing and web-based tools. Nucleic Acids Research. 2013;41: D590–D596. doi: 10.1093/nar/gks1219 23193283 PMC3531112

[43] R core team. R: A language and environment for statistical computing. R Foundation for Statistical Computing. 2021.

[44] McMurdie PJ, Holmes S. phyloseq: An R Package for Reproducible Interactive Analysis and Graphics of Microbiome Census Data. PLOS ONE. 2013;8: e61217. doi: 10.1371/journal.pone.0061217 23630581 PMC3632530

[45] Oksanen J, Blanchet FG, Kindt R, Legendre P, Minchin P, O’Hara B, et al. Vegan: Community Ecology Package. R Package Version 22–1. 2015;2: 1–2.

[46] Robinson MD, McCarthy DJ, Smyth GK. edgeR: a Bioconductor package for differential expression analysis of digital gene expression data. Bioinformatics. 2010;26: 139–140. doi: 10.1093/bioinformatics/btp616 19910308 PMC2796818

[47] Wickham H. ggplot2: Elegant Graphics for Data Analysis. Springer International Publishing; 2016.

[48] McMurdie PJ, Holmes S. Waste Not, Want Not: Why Rarefying Microbiome Data Is Inadmissible. PLOS Computational Biology. 2014;10: e1003531. doi: 10.1371/journal.pcbi.1003531 24699258 PMC3974642

[49] Simpson EH. Measurement of Diversity. Nature. 1949;163: 688–688. doi: 10.1038/163688a0

[50] Pielou EC. The measurement of diversity in different types of biological collections. Journal of Theoretical Biology. 1966;13: 131–144. doi: 10.1016/0022-5193(66)90013-0

[51] Spellerberg IF, Fedor PJ. A tribute to Claude Shannon (1916–2001) and a plea for more rigorous use of species richness, species diversity and the ‘Shannon—Wiener’ Index. Global Ecology and Biogeography. 2003;12: 177–179. doi: 10.1046/j.1466-822X.2003.00015.x

[52] Lozupone C, Lladser ME, Knights D, Stombaugh J, Knight R. UniFrac: an effective distance metric for microbial community comparison. ISME J. 2011;5: 169–172. doi: 10.1038/ismej.2010.133 20827291 PMC3105689

[53] Anderson MJ. Permutational Multivariate Analysis of Variance (PERMANOVA). Wiley StatsRef: Statistics Reference Online. John Wiley & Sons, Ltd; 2017. pp. 1–15.

[54] Anders S, Huber W. Differential expression analysis for sequence count data. Genome Biology. 2010;11: R106. doi: 10.1186/gb-2010-11-10-r106 20979621 PMC3218662

[55] Benjamini Y, Hochberg Y. Controlling the False Discovery Rate: A Practical and Powerful Approach to Multiple Testing. Journal of the Royal Statistical Society: Series B (Methodological). 1995;57: 289–300. doi: 10.1111/j.2517-6161.1995.tb02031.x

[56] Torres-Beltrán M, Mueller A, Scofield M, Pachiadaki MG, Taylor C, Tyshchenko K, et al. Sampling and Processing Methods Impact Microbial Community Structure and Potential Activity in a Seasonally Anoxic Fjord: Saanich Inlet, British Columbia. Front Mar Sci. 2019;6. doi: 10.3389/fmars.2019.00132

[57] Allgaier M, Grossart H-P. Seasonal dynamics and phylogenetic diversity of free-living and particle-associated bacterial communities in four lakes in northeastern Germany. Aquatic Microbial Ecology. 2006;45: 115–128. doi: 10.3354/ame045115

[58] Eloe EA, Shulse CN, Fadrosh DW, Williamson SJ, Allen EE, Bartlett DH. Compositional differences in particle-associated and free-living microbial assemblages from an extreme deep-ocean environment. Environmental Microbiology Reports. 2011;3: 449–458. doi: 10.1111/j.1758-2229.2010.00223.x 23761307

[59] Hahn MW, Lünsdorf H, Wu Q, Schauer M, Höfle MG, Boenigk J, et al. Isolation of novel ultramicrobacteria classified as actinobacteria from five freshwater habitats in Europe and Asia. Appl Environ Microbiol. 2003;69: 1442–1451. doi: 10.1128/AEM.69.3.1442-1451.2003 12620827 PMC150105

[60] Kim S, Kang I, Seo J-H, Cho J-C. Culturing the ubiquitous freshwater actinobacterial acI lineage by supplying a biochemical ‘helper’ catalase. The ISME Journal. 2019;13. doi: 10.1038/s41396-019-0432-x 31073214 PMC6775976

[61] Stabel H-H. Calcite precipitation in Lake Constance: Chemical equilibrium, sedimentation, and nucleation by algae1. Limnology and Oceanography. 1986;31: 1081–1094. doi: 10.4319/lo.1986.31.5.1081

[62] Küchler-Krischun J, Kleiner J. Heterogeneously nucleated calcite precipitation in Lake Constance. A short time resolution study. Aquatic Science. 1990;52: 176–197. doi: 10.1007/BF00902379

[63] Pulvermüller AG, Kleiner J, Mauser W. Calcite patchiness in Lake Constance as viewed by LANDSAT-TM. Aquatic Science. 1995;57: 338–349. doi: 10.1007/BF00878397

