# Supplementary figures and images for: Description of a ‘plankton filtration bias’ in sequencing-based bacterial community analysis and of an Arduino microcontroller-based flowmeter device that can help to resolve it
Source: PLoS One. 2024 May 28;19(5):e0303937. doi: 10.1371/journal.pone.0303937 (PMC11132488; doi:10.1371/journal.pone.0303937)

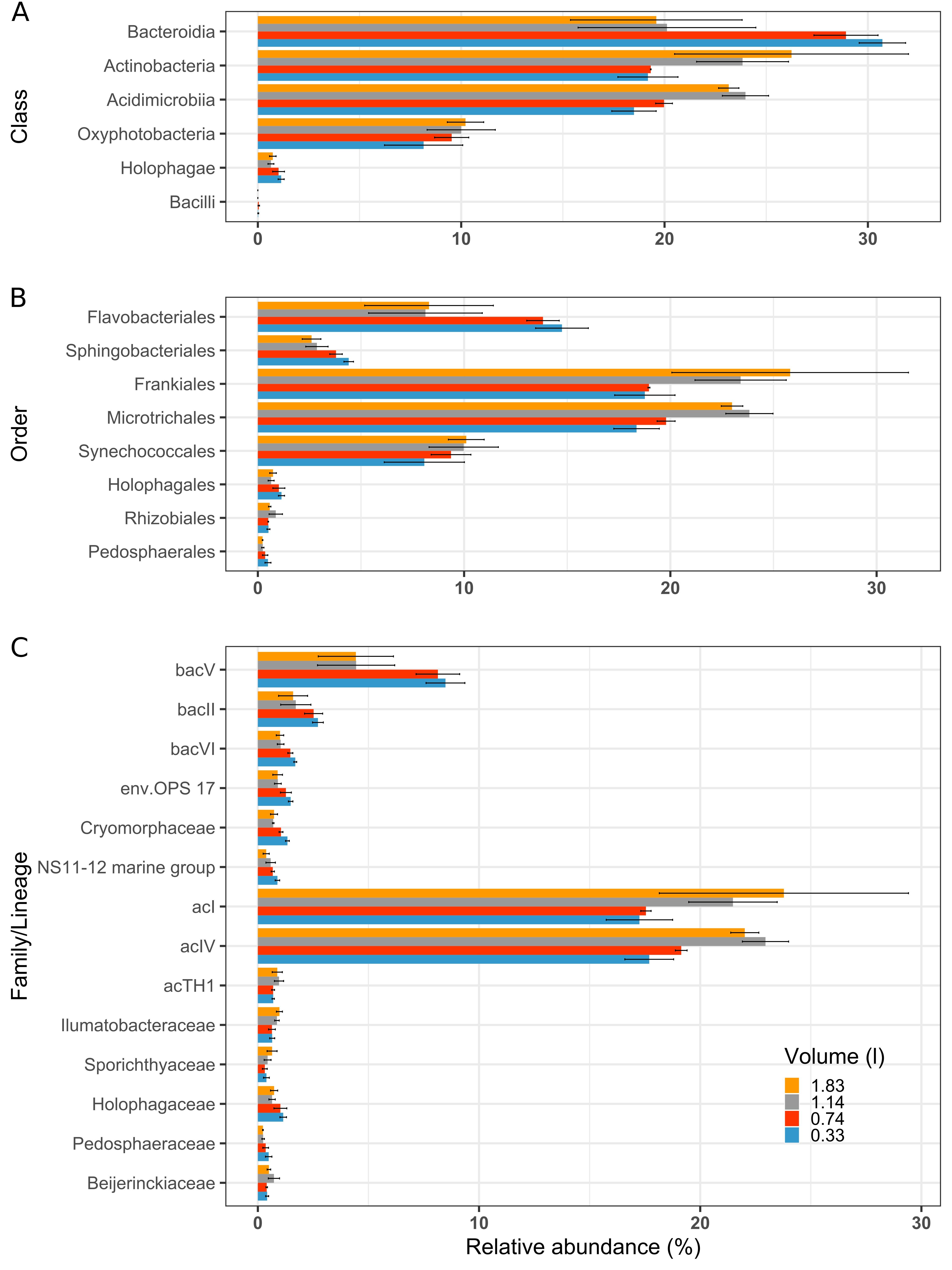

Supplement: S3 Fig — (TIF) [file pone.0303937.s003.tif]

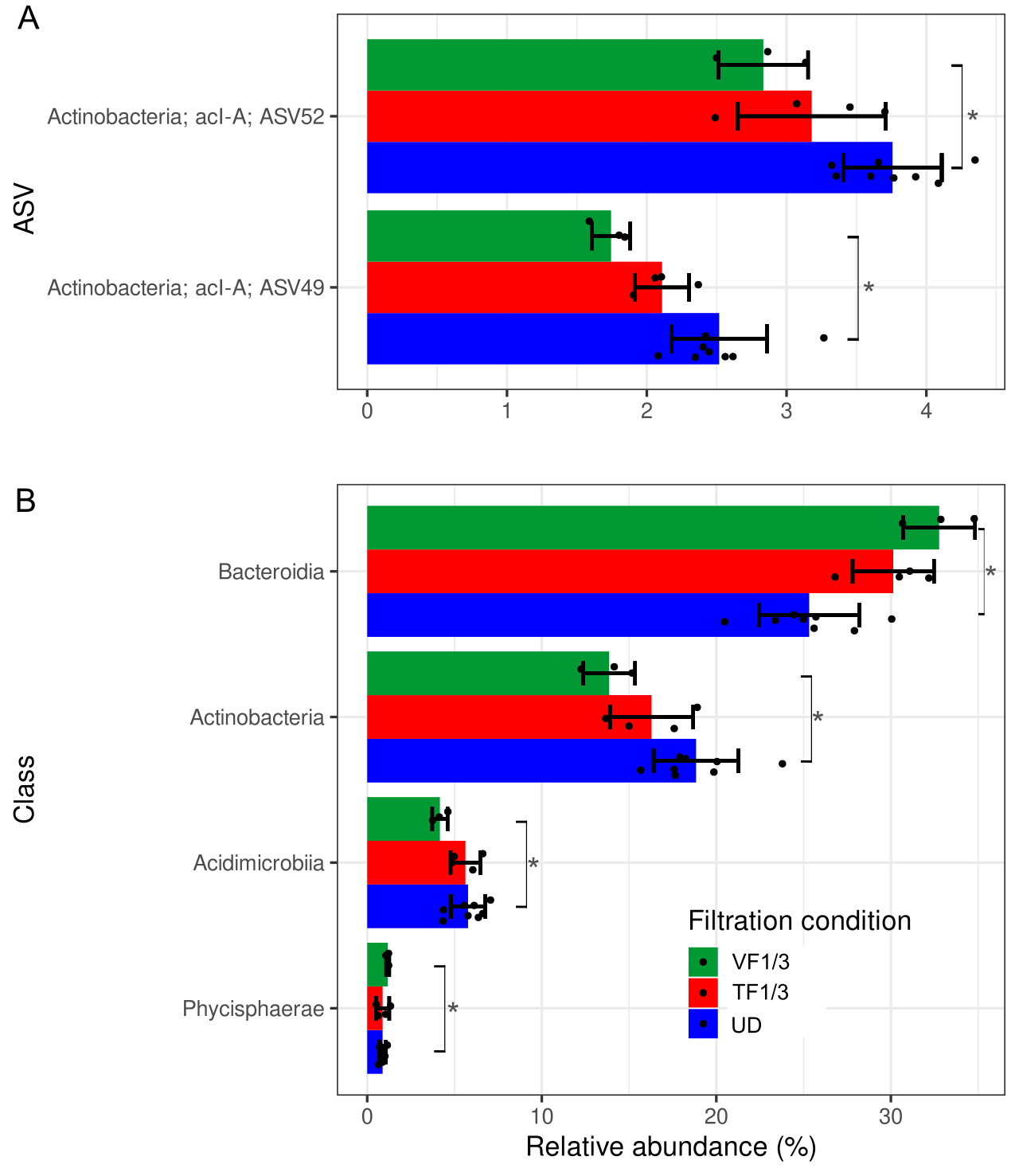

Supplement: S4 Fig — (TIF) [file pone.0303937.s004.tif]

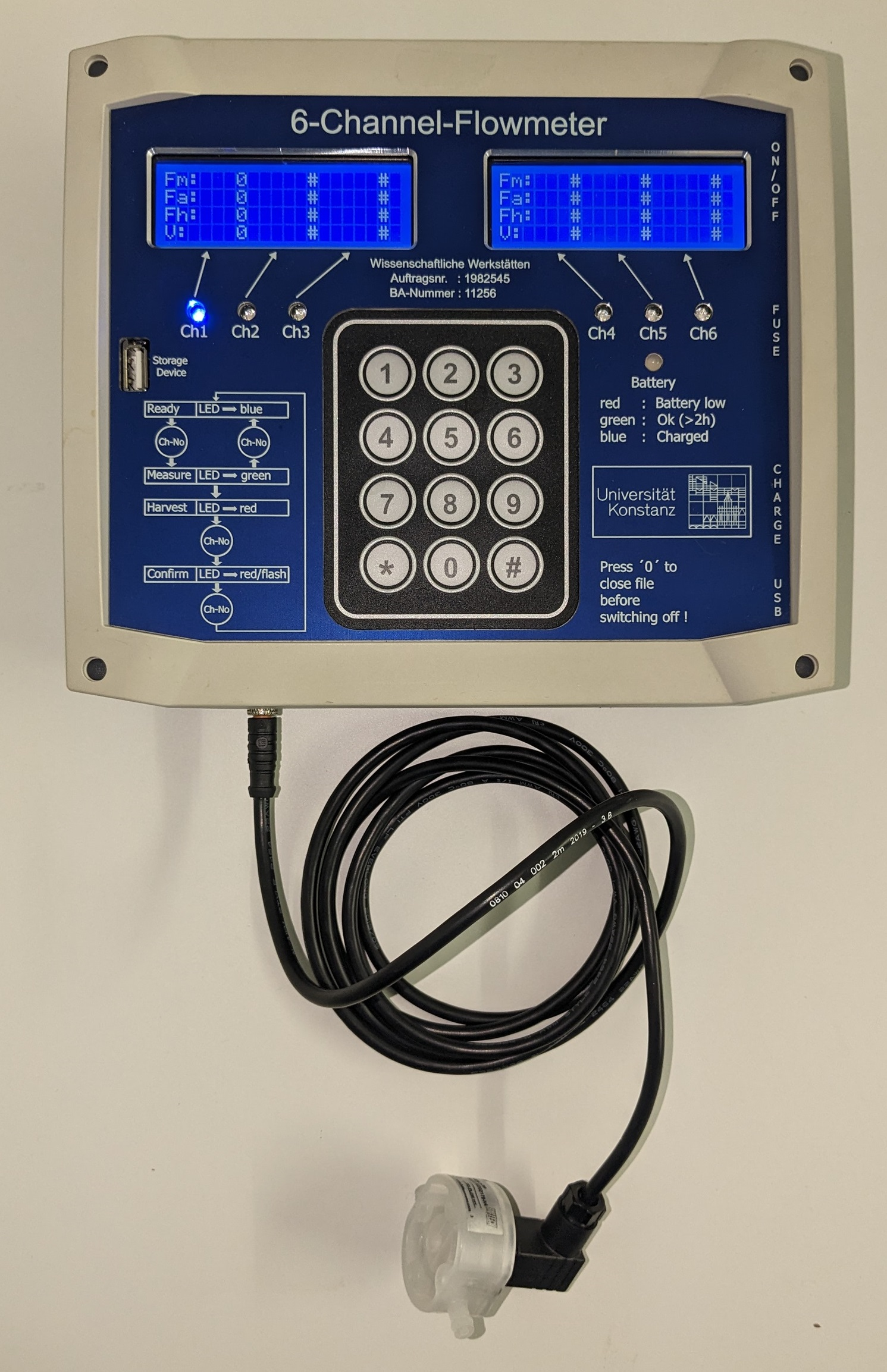

Supplement: S5 Fig — (TIF) [file pone.0303937.s005.tif]
